# Functional Nanocarriers for Delivering Itraconazole Against Fungal Intracellular Infections

**DOI:** 10.3389/fphar.2021.685391

**Published:** 2021-06-28

**Authors:** Susana P. Mejía, Arturo Sánchez, Viviana Vásquez, Jahir Orozco

**Affiliations:** ^1^ Max Planck Tandem Group in Nanobioengineering, University of Antioquia, Medellín, Colombia; ^2^ Experimental and Medical Micology Group, Corporación para Investigaciones Biológicas (CIB), Medellín, Colombia

**Keywords:** intracellular infection, poly (lactic acid-co-glycolic acid), itraconazole, nanocarriers, macrophages, bioreceptor

## Abstract

Infectious diseases caused by intracellular microorganisms represent a significant challenge in medical care due to interactions among drugs during coinfections and the development of resistance in microorganisms, limiting existing therapies. This work reports on itraconazole (ITZ) encapsulated into functional polymeric nanoparticles for their targeted and controlled release into macrophages to fight intracellular infections. NPs are based on poly (lactic acid-co-glycolic acid) (PLGA) polymers of different compositions, molecular weights, and lactic acid–to–glycolic acid ratios. They were self-assembled using the high-energy nanoemulsion method and characterized by transmission electron microscopy, Fourier transform infrared spectroscopy (FT-IR), and differential scanning calorimetry. It was studied how the polymer-to-drug ratio, changes in the aqueous phase pH, and type and concentration of surfactant affected nanocarriers’ formation, drug-loading capacity, and encapsulation efficiency. Results showed that drug-loading capacity and encapsulation efficiency reached 6.7 and 80%, respectively, by lowering the pH to 5.0 and using a mixture of surfactants. Optimized formulation showed an initial immediate ITZ release, followed by a prolonged release phase that fitted better with a Fickian diffusion kinetic model and high stability at 4 and 37°C. NPs functionalized by using the adsorption and carbodiimide methods had different efficiencies, the carbodiimide approach being more efficient, stable, and reproducible. Furthermore, linking F4/80 and mannose to the NPs was demonstrated to increase J774A.1 macrophages’ uptake. Overall, *in vitro* assays showed the nanosystem’s efficacy to eliminate the *Histoplasma capsulatum* fungus and pave the way to design highly efficient nanocarriers for drug delivery against intracellular infections.

## Introduction

Limited effectiveness of conventional therapies to combat intracellular infectious agents is commonly related to phagocytic evasion mechanisms, low-specificity treatments, manipulation of intracellular machinery by the pathogen, and the appearance of coinfections and drug resistance (Abushaheen et al., 2020). In this context, nanoencapsulation of therapeutic principles in NPs has been introduced as a powerful alternative to improve therapeutic efficacy and provide higher specificity, reducing doses and adverse effects (Begines et al., 2020; Eleraky et al., 2020; Sánchez et al., 2020). The most commonly explored nanoencapsulation systems are based on nanoemulsions, lipid NPs, natural and synthetic polymeric nanocarriers, nanocapsules, nanogels, liposomes, niosomes, etc. (Endsley and Ho, 2012; Li et al., 2017; Bentz and Savin, 2018; George et al., 2019; Colorado et al., 2020; Sánchez et al., 2020). Among them, PLGA-based polymeric nanosystems enjoy high biocompatibility and biodegradability and high reproducibility and scalability. NPs fulfill the functions of reservation and protection of the active principle. They can be designed to cross biological barriers and functionalized with targeting ligands (Mena-Giraldo et al., 2020; Sánchez et al., 2020) at the outermost NP surface for enhanced cellular uptake, releasing the therapeutic load in a site-specific manner. Functional nanocarriers have been demonstrated to have the potential for the management of intracellular infections caused by viruses, certain bacteria (e.g., *Mycobacterium tuberculosis*), some protozoa (e.g., *Toxoplasma gondii* and *Leishmania* spp.), and some fungi (e.g., *Histoplasma capsulatum*) (Eleraky et al., 2020; Sánchez et al., 2020).

The size, shape, physicochemical properties, and surface chemistry of NPs are tuned on demand for a specific purpose (Duan and Li, 2013; Sánchez et al., 2020). One of the most significant challenges in developing functional nanocarriers is their interaction with physiological proteins, which may form a biomolecular corona (or protein corona), changing their superficial properties, affecting their circulation time in the body, and decreasing their specificity and effectiveness. The protein corona impacts antibody-coated NPs’ targeting and may depend on the functionalization method owing to their partial or total NP masking (Mahmoudi et al., 2016; Tonigold et al., 2018). Antibodies may bind to the NPs reversibly through ionic, electrostatic, hydrophobic, or Van der Waals interactions (Tonigold et al., 2018) or irreversibly through covalent bonds. Interactions depend not only on NPs’ affinity, composition, and pending functional groups but on antibody isoelectric point, surface chemistry, and pH conditions. Although physical adsorption may show low reproducibility and low stability at different pH conditions, good results have been reported regarding antibodies’ orientation (Oliveira et al., 2019). Covalent coupling depends on the NPs’ surface and ligand reactive groups. Linking ligands to NPs by covalent bonding, such as carbodiimide chemistry, offers high stability despite possible aggregation, polymerization, and random antibody orientation, thus affecting the accessibility of the antigen-binding sites (Oliveira et al., 2019).

**SCHEME 1:**
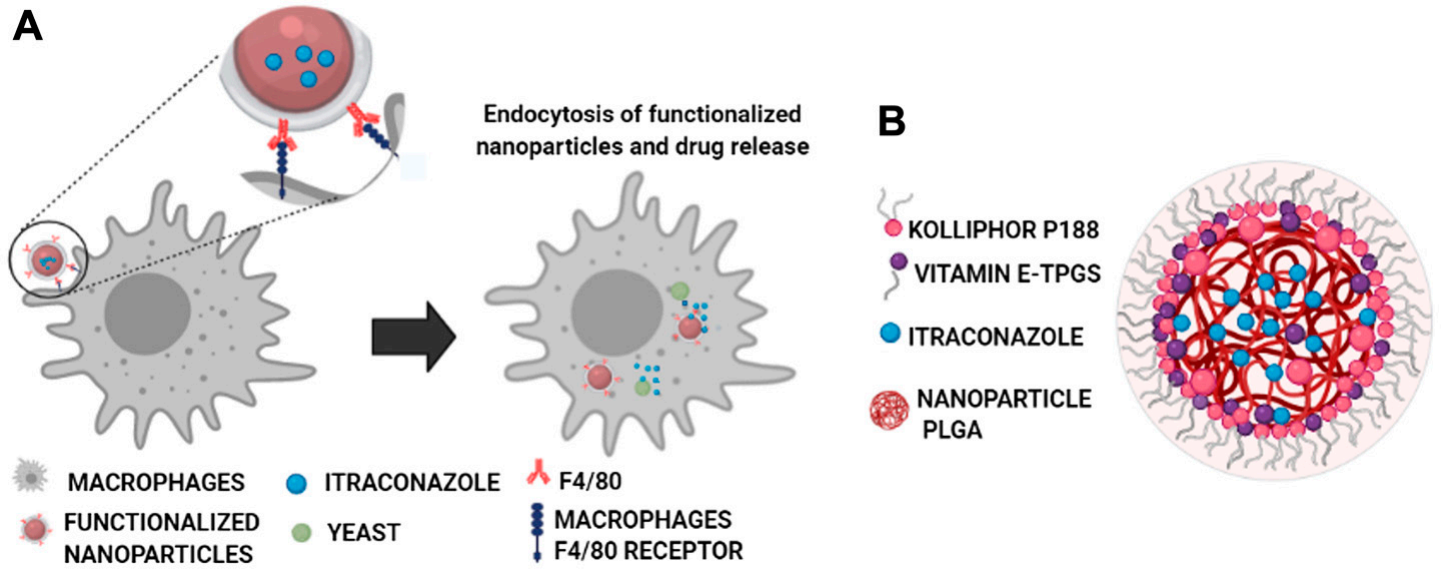
Functionalized NPs’ specific interaction of the F4/80 antibody (in red) with macrophages’ F4/80 receptor (in blue) through non-covalent interactions (hydrogen bonding, Van der Waals, and hydrophilic interactions) with the N-terminal region of the receptor, composed of six EGF-like domains (Lin et al., 2010) **(A)**, and core–shell–like type schematic representation of the components of the self-assembled nanocarriers with the optimized ITZ-PLGA-TPGS-pH5 formulation without the targeting ligand **(B)**.

This work aims to develop a new therapeutic strategy based on nanoencapsulation of a therapeutic agent into NPs with high specificity toward macrophages to fight intracellular infections (Scheme 1A), with antifungal itraconazole (ITZ) as a hydrophobic drug model (Scheme 1B). This drug is commonly prescribed to patients with serious fungal infections such as histoplasmosis, an infection produced by the etiological agent *H. capsulatum.* This fungus, together with *Pneumocystis* spp, is currently associated with the development of coinfections in HIV-positive patients, whose presence in some geographical regions is even higher than that of bacteria such as *M. tuberculosis* (Agudelo et al., 2012; Caceres et al., 2018; Carreto-Binaghi et al., 2019). By encapsulating ITZ in NPs, we expect to reduce the limitations related to its high lipophilicity and low absorption ability (Ling et al., 2016; Alhowyan et al., 2019; Biswaro et al., 2019). ITZ nanoformulations include nanocrystals (Wan et al., 2018), NPs (Alhowyan et al., 2019; Jana et al., 2019), and solid lipid nanoparticles (Kim et al., 2010), among others (Hong et al., 2006; Chen et al., 2008; Sharma et al., 2016; Karashima et al., 2017). However, few studies have addressed directing functionalized NPs toward macrophages. The current study develops a biocompatible formulation of ITZ encapsulated into PLGA NPs with optimal colloidal properties regarding size, moderate polydispersity, and surface charge and optimal DLC and EE for adequate ITZ release (Scheme 1B). NPs were further functionalized with the F4/80 antibody and mannose by physical adsorption and chemical coupling for targeted cargo delivery into macrophages (Scheme 1A), demonstrating efficacy in eliminating the *H. capsulatum* fungus. To the best of our knowledge, this is the first report showing that F4/80-functionalized NPs can help improve macrophage-targeted therapy and with similar efficiency to that of mannose-coupled NPs. Therefore, functional nanocarriers could be a platform for drug encapsulation as a promising therapeutic alternative to fight infectious diseases.

## Materials and Methods

### Chemicals

Poly (lactic acid-co-glycolic acid) (PLGA); LA:GA 75:25 (RG 752H) with an inherent viscosity of 0.14–0.22 dl/g (4–15 kDa) and PLGA; LA:GA 50:50 (RG 503H) with an inherent viscosity of 0.32–0.44 dl/g (24–38 kDa) were generously donated by Evonik (Essen, Germany). Sigma-Aldrich provided Nile red (CAS 7385-67-3), itraconazole (ITZ, CAS 84625-61-6), poloxamer 188 (CAS 9003-11-6, Kolliphor®), D-α-tocopherol polyethylene glycol 1,000 succinate (vitamin E-TPGS, CAS 9002-96-4), voriconazole (CAS 137234-62-9), and phosphate buffer saline (PBS D8573). D-mannose (CAS 3458-28-4), Kit MTT TOX-1, HMM broth (nutrient media F12 HAM, N6760), Janus Green (CAS 2869-83-2), tween 80 (CAS 9005-65-6), sodium periodate (CAS 7790-28-5), Hoechst (CAS 23491-45-4), and ethylenediamine (EDA, CAS 107-15-3) were purchased from Sigma-Aldrich (St Louis, MO, United States). Dulbecco’s modified Eagle medium (DMEM, Ref. 10569010) and penicillin–streptomycin (Ref. 15140122) were purchased from Gibco (Gibco, Thermo Fisher Scientific, Inc., Waltham, MA, United States). Ethyl acetate (CAS 141-78-6), ethanol (CAS 64-17-5), acetonitrile (CAS 75-05-8), and dimethyl sulfoxide (DMSO, CAS 67-68-5) were bought from Merck. Sucrose (CAS 57-50-1) and citric acid (CAS 77-92-9) were purchased from VWR Chemicals. Sodium chloride (CAS 7647-14-5), potassium chloride (CAS 7447-40-7), and potassium phosphate monobasic (CAS 7778-77-0) were provided by J. T. Baker (J.T. Baker, Thermo Fisher Scientific, Inc., Waltham, MA, United States). Sodium citrate dihydrate (CAS 6132-04-3) and sodium phosphate dibasic (CAS 7558-79-4) were acquired from Panreac. F4/80 antibody (ab100790), IgG isotype (ab171870), and Alexa flour 488 (ab150077) were obtained from Abcam (Abcam, Cambridge, United Kingdom). Brain–heart infusion (BHI) was obtained from Difco Laboratories (Difco Laboratories, Ref. DF0418, Thermo Fisher Scientific, Inc., Waltham, MA, United States). Fetal bovine serum (FBS) was acquired from Trynyty TEK (01010102, Cartagena, CO). 0.1 M citrate buffer (pH 4.5–5.5), 50 mM MES buffer (pH 6.0–6.5), 50 mM HEPES buffer (pH 7.0), and 0.1 M PBS buffer (pH 6.5) were prepared by dissolving the reagents as received in Milli-Q water and filtrating using a 0.2-µm filter.

### Microorganisms and Cell Lines

*Histoplasma capsulatum* strain CIB 1980 was isolated from a human clinical case and deposited in the collection of the Experimental and Medical Mycology Group, CIB, Medellín-Colombia. Yeasts were grown in BHI broth and supplemented with 0.1% l-cysteine and 1% glucose at 37°C with aeration on a mechanical shaker (150 rpm, Innova® 44, Thermo Fisher Scientific, Inc., Waltham, MA, United States) and routinely collected during the exponential growth phase (at 48 h). Fungal growth was collected and passed 20 times through a tuberculin syringe with a 1/2-inch needle to eliminate clumped fungal cells. Viability and number of yeasts were determined by staining with Janus Green. The fungal unit was considered to be equivalent to a single cell or mother cell with 2–7 daughter cells. Fungal cells were counted in a hemocytometer and resuspended in HMM culture medium to obtain the desired number. Murine macrophages J774A.1 were obtained from the American Type Culture Collection (ATCC, Manassas, VA, United States). Cells were cultured in suspension in DMEM medium supplemented with 10% FBS and 1% penicillin–streptomycin at 37°C with 5% CO_2_. Cells were passaged every 2–3 days. Viability was determined by visual microscopic inspection of the nuclei stained with trypan blue.

### Nanoemulsion Formulations

NPs were prepared using the high-energy emulsification method using a variety of PLGA polymer compositions, that is, different glycolic acid–to–lactic acid ratios (50:50 or 75:25) and molecular weights (24–38 and 7–14 kDa, respectively), namely, Resomer® RG 503H and RG 752H. Briefly, 75 mg of PLGA was dissolved in 5 ml ethyl acetate, and the organic phase was poured into 10 ml 1% Kolliphor® P188 solution under vortex mixing at 3,200 rpm for 20 s. Immediately after mixing, the microemulsion was subjected to three ultrasonication pulses using a 1/8-inch probe at 20% amplitude (Ultrasonicator Q500, Qsonica, Newton, CT, United States) to make the nanoemulsion. Afterward, the nanoemulsion was put in a rotary evaporator under 300 mbar at 150 rpm for 10 min at 40°C (Rotavapor® R-300, Buchi, Germany). The resulting NPs were purified by dialysis with 20% ethanol. Finally, the colloidal dispersion in ultrapure water was lyophilized. To obtain NPs with the proper size, surface charge, drug-loading capacity, and release kinetic, preliminary studies were carried out with various surfactants either in the aqueous or in the organic phases. Table 1 summarizes the colloidal properties of the formulations studied.

**TABLE 1:** Colloidal properties of PLGA NPs with encapsulated ITZ.

Polymer (PLGA)	ITZ (mg)	Size (nm)	PDI	ζ-potential (mV)	DLC (%)	EE (%)
50:50	3	189.9 ± 7.5	0.21 ± 0.01	−50.7 ± 3.3	2.8 ± 0.1	72.1 ± 0.2
	6.6	188.5 ± 3.0	0.23 ± 0.04	−39.9 ± 2.0	4.0 ± 0.2	47.6 ± 3.2
	7.5	272.5 ± 43.3	0.41 ± 0.12	−46.4 ± 2.4	8.2 ± 0.2	89.4 ± 2.8
75:25	6.6	150.4 ± 6.4	0.16 ± 0.02	−40.9 ± 2.9	6.0 ± 0.2	75.2 ± 2.9
50:50-pH5	6.6	217.7 ± 5.5	0.24 ± 0.04	−46.9 ± 1.3	5.6 ± 0.1	68.0 ± 0.3
50:50-TPGS-pH5	6.6	165.0 ± 9.7	0.23 ± 0.05	−38.9 ± 2.1	6.4 ± 0.7	78.3 ± 8.6
75:25-TPGS	6.6	157.7 ± 7.7	0.16 ± 0.01	−38.8 ± 2.1	6.1 ± 1.1	75.0 ± 13.0
75:25-TPGS-pH5	6.6	147.3 ± 7.7	0.19 ± 0.01	−38.0 ± 0.3	6.7 ± 1.3	80.1 ± 11.8

### Encapsulation of Model Hydrophobic Compounds Into NPs

Three different polymer-to–active ingredient ratios (1:25, 1:11, and 1:10) were evaluated to encapsulate ITZ in PLGA NPs, which allowed the determination of how the ITZ concentration influenced its encapsulation process. The emulsification method was used as described above. 3.0–7.5 mg ITZ was dissolved in 5 ml of ethyl acetate. A certain quantity of freeze-dried NPs was weighed, dissolved in acetonitrile, and analyzed using a validated HPLC chromatography method in the reverse phase using a C18 column (Eclipse XDB, 150 × 4.6 mm, 5 µm), a UV-VWD detector, voriconazole as internal standard, a 40:60 ratio of water to acetonitrile mobile phase, isocratic mode at 1 ml/min, and a wavelength of 261 nm (Cáceres et al., 2016) to find out the amount of ITZ loaded into the NPs. As described above, the emulsification method was used to encapsulate Nile red as a model molecule (at a concentration of 120 µM). Quantification of encapsulated Nile red was evaluated by UV–visible spectrophotometry at 520 nm.

### Determination of the Encapsulation Efficiency and Drug-Loading Capacity

Determination of DLC and EE in NR-PLGA-NPs and ITZ-PLGA-NPs, respectively, was estimated from the following:

$$\text{DLC}=\frac{\text{Encapsulated mass}\;\left(\text{NR or ITZ}\right)\;\text{into nanoparticles}\;\left(\text{mg}\right)\times \;100\%}{\text{Total mass of nanoparticles}\;\left(\text{mg}\right)},$$

$$\text{EE}=\;\frac{\text{Encapsulated mass }\;\left(\text{NR or ITZ}\right)\;\text{into nanoparticles }\;\left(\text{mg}\right)\times \;100\%}{\text{Mass }\;\left(\text{NR or ITZ}\right)\text{ initially added }\;\left(\text{mg}\right)}.$$

### Physicochemical Characterization of Nanoparticles

NP size and size distribution (PDI) were obtained by dynamic light scattering (DLS) and *ζ*-potential (mV) was obtained by electrophoretic light scattering (ELS) in a Zetasizer-pro (Malvern Instruments, United Kingdom) at 25°C after adequate aqueous dilution in triplicate. Transmission electron microscopy (TEM) determined the morphology of the NPs. A drop of the nanoparticle dispersion was added to a copper grid with a carbon membrane and allowed to dry at room temperature to obtain the TEM images (Tecnai F20 Super Twin, FEI). Digital micrograph software was used for image treatment. Thermal analysis was performed by differential scanning calorimetry (DSC) using a TA Instruments Q20 model. The analysis was carried out with the lyophilized nanoparticles loading itraconazole and its precursors, causing thermal deletion before analyzing each sample. The method consisted of weighing 4- to 8-mg samples, sealing them in a hermetic aluminum capsule, and analyzing at temperatures ranging from 20 to 350°C at 10°C/min in a nitrogen atmosphere to obtain the respective thermograms. NPs with the encapsulated drug, functionalized nanoparticles, physical mixture of the precursors, and precursors alone were characterized by FT-IR spectroscopy using the transmittance accessory in Thermo iS50 equipment. Materials were prepared as potassium bromide (KBr) pellets, and the measurements were carried out using 32 scans with a resolution of 4 cm^−1^. Spectra were collected from 4,400 to 350 cm^−1^ wavenumbers. Before the analysis, the baseline was measured under the same conditions using a pure KBr tablet as a reference.

### Drug Release and Stability

For evaluating the ITZ release profile, 3 ml dilution of the original solution of fresh NPs was prepared, diluted in 25 ml of sterile purified water, and centrifuged at 10000 rpm and 4°C for 30 min. After this time, the supernatant was discarded, and the pellet was resuspended in 25 ml of release medium simulating physiological conditions (PBS solution at pH 7.2) containing 1% v/v of tween 80. 1.0 ml of this solution was added to each 1.5-ml tube for a total of 11 analysis times (0, 0.5, 1, 2, 3, 4, 6, 7, 24, 48, and 72 h, respectively). Subsequently, the tubes were incubated in a thermal mixer at 800 rpm and 37°C, and each vial was removed at the selected times. Then, the vials were centrifuged at 10,000 rpm and 25°C for 1 h. The supernatant and the pellet were used for the direct and indirect determination of ITZ by HPLC chromatography.

The stability of NPs from two types of polymers was evaluated after lyophilization and cryopreservation with 5% sucrose. 4 and 25°C were studied as storage temperatures for up to one month. The dispersion of optimized NPs stored at 4°C without lyophilization for one month was also evaluated. Additionally, the stability of dispersion at 37°C was analyzed under the same kinetic release conditions (PBS pH 7.2, tween 1% v/v at 37°C). For this purpose, 20 mg of the NPs was added to 1.5-ml tubes, and the samples were extracted at the beginning and at least five times between the first and fourth evaluation weeks. The lyophilized and dispersed NPs were resuspended in 1 ml of sterile purified water, and size, surface charge, and PDI were evaluated as mentioned above.

### Antifungal Susceptibility Testing

The antifungal activity of ITZ loaded into PLGA-NPs was compared against ITZ–aqueous suspensions. The fungal growth inhibition was tested using the microdilution method for yeast (M27-A3) following the National Committee for Clinical Laboratory Standards (NCCLS), with empty PLGA-NPs and each component of the NPs (PLGA, TPGS, and Kolliphor P188) as a control. Yeast suspensions were prepared in HMM medium adjusted to a concentration of 3 × 10^5^ CFU. Aliquots of 0.1 ml of suspension yeast were added per well. The dilutions of each treatment were prepared in HMM medium with 1% DMSO. Final drug concentrations were 16–0.007 μg/ml for each treatment, using the medium with 1% of solvent without the drug as the control. The plate was incubated at 37°C with aeration on a mechanical shaker (150 rpm, Innova® 44, Thermo Fisher Scientific, Inc., Waltham, MA, United States) for 7 days. When the incubation was finished, the fungal growth was checked by visible turbidity, and fungus viability was determined through the 3-(4,5-dimethylthiazol-2-yl) 2,5-diphenyltetrazolium bromide (MTT) method. All experiments were performed in triplicate.

### Immobilization of Antibodies on Poly (Lactic Acid-Co-Glycolic Acid) NPs and Mannose-Coated NPs

Functionalization of NPs with antibodies by physical adsorption and covalent coupling were compared in terms of efficiency. For the physical immobilization of the antibodies on the surface of the PLGA75:25-TPGS-pH5 NPs with encapsulated Nile red, 25 μl of the particle dispersion (1.5 mg/ml in 0.1 M citrate buffer with a pH value between 4.5 and 5.5, 50 mM MES buffer with a pH value between 6.0 and 6.5, 0.1 M PBS with a pH value of 6.5, or 50 mM HEPES buffer with a pH value of 7.0) was diluted with 4 ml of MES buffer. The NP dispersion was poured dropwise into 0.36 mg/ml anti-F4/80 antibody, depending on the 1:5, 1:10, and 1:20 ratios, dissolved in 50 or 300 μl of the buffer according to the pH to be evaluated. This solution was incubated at room temperature for 4 or 12 h or at 4°C for 24 h under constant shaking. A button of functionalized NPs was obtained by ultracentrifugation at 10,000 rpm at 4°C for 30 min, followed by two washing steps with 600 μl of water by 10,000-rpm ultracentrifugation at 4°C for 30 min. The final button was diluted to a final volume of 17 μl with sterile 1X PBS.

The covalent attachment of antibodies to the surface of PLGA NPs was by the EDC/NHS coupling reaction, using a two-step process involving the activation of a carboxyl group and subsequent conjugation with primary amines. PLGA75:25-TPGS-pH5 NPs with encapsulated Nile red were diluted to 1.5 mg/ml in 50 mM MES buffer (pH 6.5). 23 mg (100 mM) of NHS and 153 mg (400 mM) of EDC-HCl were dissolved in 1 ml 50 mM MES buffer (pH 6.5), added to 1 ml of this dispersion, and shaken at 800 rpm at room temperature for 30 min. The reaction mixture was then washed three times with MES buffer by centrifugation at 10,000 rpm for 30 min to remove the unreacted material and diluted to a final volume of 2 ml with 10 mM PBS (pH 6.5). 5.7 μl of the activated NP dispersion (6.5 mg/ml) was diluted in 50 μl PBS (pH 6.5). 10.4 μl of F4/80 (0.36 mg/ml) or 3.75 μl of mouse IgG (1 mg/ml) were dissolved in 50 μl of PBS. The NP dispersion was added dropwise to the antibody solution, and the combined reaction mixture was shaken at 4°C for 2 h. The functionalized NPs were then washed twice with 10 mM PBS (pH 7.2) by centrifugation at 3,000 rpm for 30 min. The final volume of the dispersion was adjusted to 17 μl with 10 mM PBS (pH 7.2).

Mannose-covered NPs were prepared based on previous works (Ghotbi et al., 2011), with modifications. Briefly, 3 mg of each PLGA NP was resuspended in 2 ml of MES (pH 6.5) and mixed with 500 μl 0.1 M NHS and 500 μl 0.4 M DCC. After 30 min, 0.2 μl of EDA was added and incubated for 2 h. Simultaneously, the D-mannose ring was opened by treating 3 mg of it with 10 mM NaIO_4_ solution and incubated under constant agitation for 30 min at room temperature. Then, the mannose was added to the solution with PLGA NPs and incubated for 12 h at room temperature (RT) under agitation at 800 rpm. Afterward, the NPs were washed with 10 mM PBS through centrifugation at 10,000 rpm at 4°C for 30 min thrice to remove the excess EDA and unreacted mannose.

### Detection of Antibodies on NPs

4, 10, 20, and 30 μg/ml goat antiRabbit IgG Alexa Fluor 488 (AF 488) were added to the solution of functionalized NPs. 1X sterile PBS was added until the final volume of 20 μl was reached. This solution was incubated at room temperature for 30 min under constant shaking. Once the incubation was finished, it was brought to a final volume of 200 μl to measure them by flow cytometry or fluorescence spectrophotometry, respectively. A calibration curve was made with different concentrations of AF488 between 25 and 0.195 μg/ml, the fluorescence intensity values were plotted against concentration (Supplementary Figure S4A,S4B), and the values of the unknown antibody concentrations were calculated from the straight-line equation.

### Study of Protein Corona Formation

The functionalized NPs were incubated with 10–100% fetal bovine serum at 37°C for 1 h to allow protein adsorption in a final volume of 20 μl, and then, 3.4 μl was taken to perform size and surface charge measurements as previously stated. The functionalized NPs gestated with the protein corona were incubated with 20 μg/ml of secondary AF 488 antibody at room temperature for 30 min, brought to a volume of 100 μl with sterile PBS, and measured in terms of fluorescence intensity by spectrophotometry to evaluate the accessibility of the primary F4/80 antibody after the protein corona was formed (Tonigold et al., 2018).

### *In vitro* Assay of Specificity

5 × 10^4^ J774.1A cells were adhered onto a 96-well plate for 16 h in DMEM with 10% BFS. Then, 1.5 μg/ml of functionalized NPs using the method that presented a higher degree of functionalization or uncoated NPs were added and incubated at 37°C for 3 h with 5% CO_2_. Therefore, monolayers were washed three times with PBS that was previously tempered to eliminate no endocytosed NPs. The cell cultures were characterized by fluorescence microscopy. Determination of Nile red inside the cells (area of Nile red) was measured indirectly to find out the differences in ligands’ specificity. To corroborate the NPs’ uptake, we used Hoechst dye to stain cells’ nuclei and colocalize the NPs. The area of Nile red (%) was determined using ImageJ software version 1.48 (National Institutes of Health). Ten images were captured using a 40X objective and analyzed randomly from different regions. Individual cells and Nile red areas were framed with freehand selection to measure the inner region. These areas were taken as the total area, and the area of Nile red was calculated using the Microsoft Excel 2013 package using the data (Mejía et al., 2015).

### Experimental Design and Statistical Analysis

A Plackett–Burman type screening experimental design was used randomly with three central points in duplicate, and the statistical package Statgraphics® Centurion XVII was used to evaluate the nanoemulsion method’s critical parameters to get a total of 15 tests. All statistical analyses used GraphPad Prism software (version 8.0); normal distribution was determined using ANOVA and verified using the Kolmogorov–Smirnov normality test. According to the Gaussian distribution of data, differences between groups were analyzed using Student’s t-test or the Mann–Whitney test. A *p*-value ≤ 0.05 was considered to be statistically significant.

## Results

### Preparation and Characterization of NPs

A rational Plackett–Burman experimental design screened the most important factors influencing nanocarriers’ self-assembly, using the nanoemulsion method with Nile red as a hydrophobic molecule model. The design consisted of fifteen experiments that evaluated the influence on the NPs’ size, PDI, and surface charge as response variables. The factors evaluated were concentrations of PLGA (5–15 mg/ml), Nile red (20–40 µM), and surfactant (3–5%), sonication amplitude (50–80%), time (30–40 s), and organic phase–to–water phase ratio (v/v; 1:2, 1:1.5, and 1:1). The process was studied with PLGA of two different compositions (50:50 or 75:25 glycolic acid–to–lactic acid ratio) and molecular weights (24–38 and 7–14 kDa). Optimal experimental conditions were established to assemble PLGA nanocarriers with a size of 200 nm, PDI <0.3, and *ζ*-potential ≤−30 mV, which are considered adequate for our purpose. From the experimental design, the optimal conditions were 15 mg/ml PLGA, 30 µM Nile red, 3% surfactant, 20% sonication amplitude, 30 s sonication time, and 1:2 organic-to-water phase ratio, in which the diameter of NPs from PLGA 50:50 (148.2 ± 23.3 nm) was larger than that of PLGA 75:25 NPs (119.9 ± 18.1) (Supplementary Figure S1A). This behavior is explained by the increase in the inherent viscosity of the PLGA 50:50 system (0.32–0.44 dl/g), with a higher molecular weight than that of PLGA 75:25 (0.14–0.22 dl/g). Nanocarriers from PLGA 50:50 had a particle size distribution with a slightly lower polydispersity (0.12 ± 0.02) than nanocarriers from PLGA 75:25 (0.16 ± 0.04). There were no statistically significant differences between the *ζ*-potential values, those being negative in both systems (−34.5 ± 9.0 and −32.9 ± 6.8 mV) for NPs from PLGA 50:50 and 75:25, respectively.

**FIGURE 1:**
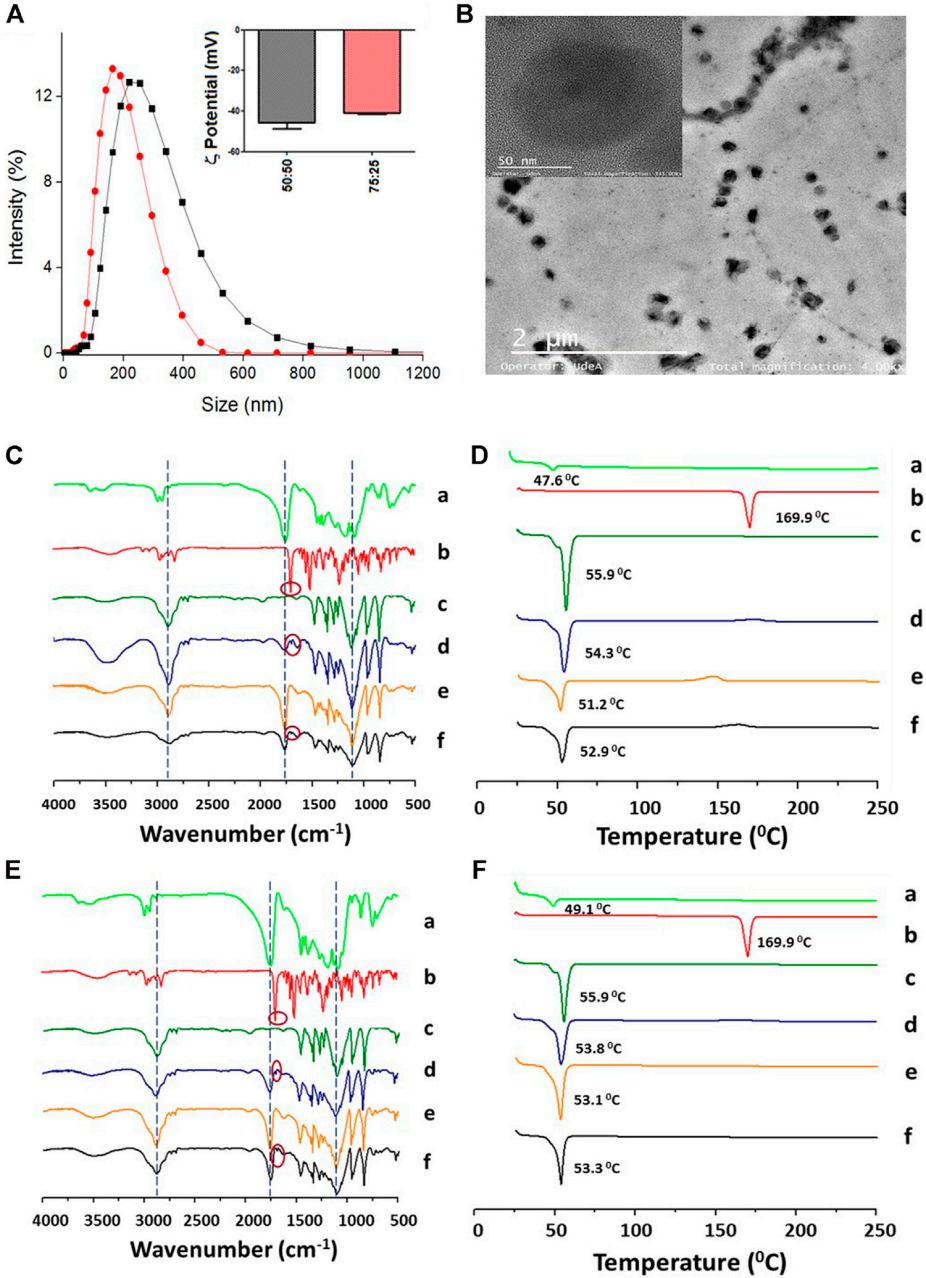
Physicochemical characterization of the PLGA NPs (base formulation) with encapsulated ITZ. **(A)** Hydrodynamic size and *ζ*-potential of PLGA 50:50 (black line and bars) and PLGA 75:25 (red line and bars). **(B)** Morphologic characterization of PLGA 50:50 NPs by TEM. **(C,** **D)** Characterization of PLGA 50:50 and **(E, F)** PLGA75:25 NPs, respectively, by FT-IR **(left)** and DSC **(right)**. PLGA (a), ITZ (b), Kolliphor P188 (c), physical mixture of 1:11:37.9 ITZ-to-PLGA-to–Kolliphor P188 weight ratio (d), and NPs from the mixture without (e) and with (f) encapsulated ITZ.

At the optimized conditions, named from now on as “base formulation,” the influence of the PLGA-to-ITZ ratio on the physicochemical properties, DLC, and EE was studied. Nanocarriers were prepared from PLGA 50:50 by changing the ITZ weight (3, 6.6, and 7.5 mg) but keeping constant the PLGA weight (15 mg/ml) to obtain different ITZ-to-PLGA ratios (1:25, 1:11, and 1:10). HPLC achieved quantification of ITZ using the isocratic method to determine DLC and EE. DLC (8.2 ± 0.2%) and EE (89.4 ± 2.8%) of the 1:10 ITZ-to-PLGA system increased significantly with respect to the 1:25 ITZ-to-PLGA system, which has a DLC of 2.8 ± 0.1% and an EE of 72.1 ± 0.2%. However, the particle size increased to 272.5 ± 43.3 nm with a PDI of 0.4 ± 0.1. Such formulation was tested with NPs of PLGA 75:25, showing proper size (150.4 ± 6.4 nm) and suitable properties (−40.9 ± 2.9 mV, PDI 0.16 ± 0.02, DLC 6.0 ± 0.2%, and EE 75.2 ± 2.9%) (Figure 1A; Table 1). The size, distribution, degree of aggregation, and morphological homogeneity of the NPs were studied by TEM analysis of air-dried unstained samples showing low and high magnification images (Figure 1B; Supplementary Figure S2A). The particle dense core image of the as-assembled NPs presented spherical and quasi-spherical shape morphology with a smooth surface, absence of vesicular structures, and low degree of aggregation or coalescence. NPs presented an average particle size of 132.0 ± 36.6 nm (PLGA 75:25) and 145.0 ± 28.2 nm (PLGA 50:50) (Figure 1B; Supplementary Figure S2A), with *n* = 30, respectively.

FT-IR and DSC characterized the nanoencapsulation process to determine ITZ–PLGA interactions. Figures 1C,E show the FT-IR spectra of the individual precursors (a–c), the physical mixture (d), and NPs without (e) and with (f) encapsulated ITZ in both types of polymers. The physical mixture shows signals mostly from the contribution of peaks at 1,767 cm^−1^ (C=O stretching) from PLGA polymers, a little peak at 1,697 cm^−1^ (C=O stretching) from the amide group of ITZ, and at 2,844 cm^−1^ (C–H stretching) and 1,106 cm^−1^ (C–O stretching) from the stabilizer. The physical mixture pattern changed in the NPs with encapsulated ITZ (f) with respect to empty NPs (e), showing a peak at 1767 cm^−1^ (C=O stretching) of higher intensity. The ITZ characteristic peaks at 1,697 cm^−1^ (C=O stretching from the amide group), 1,520 cm^−1^ (C=N stretching), and 1,232 cm^−1^ (C–N stretching) could not be observed in NPs with encapsulated ITZ, thus indicating PLGA–ITZ interactions. Figures 1D,F show the DSC analysis of the individual precursors (a–c), the physical mixture (d), and NPs without (e) and with (f) encapsulated ITZ in both types of polymers. For example, the thermograms 1 F show the glass transition temperature and the single endothermic melting peak at 49.1 and 55.9°C from the PLGA 75:25 polymer (a) and the surfactant Kolliphor P188 (c), respectively. A single-phase transition at 53.8°C was observed for the physical mixture of the components. In comparison, free ITZ (b) shows a single endothermic melting peak at 169.9°C, typical of a drug in the crystalline form, whereas empty NPs (e) display a single endothermic melting peak at 53.1°C. Remarkably, the melting peak from ITZ completely disappeared in the NP-encapsulated ITZ thermogram, thus indicating that PLGA and ITZ might be interacting through the triazole group (or the amine group) of ITZ and the carboxylic group of the PLGA hydrophobic tail (Yi et al., 2007). A similar analysis is derived from the thermogram in Figure 1D for PLGA 50:50.

The next set of experiments introduced some base formulation modifications to improve its physicochemical and structural properties, evaluated by the DLC, EE, and drug-release profile. Modifications consisted of lowering the pH of the aqueous solution to five and including a multipurpose amphiphilic excipient such as vitamin E-TPGS in a 1:10 ratio by weight with respect to PLGA, one at a time. When ITZ was encapsulated in PLGA 50:50 NPs at a pH value of 5, the DLC (5.60 ± 0.03%) and EE (68.0 ± 0.3%) increased with regard to NPs at neutral pH (Table 1), with other characteristics within the expected values (217.7 ± 5.5 nm, 0.24 ± 0.04, and −46.9 ± 1.3 mV) for size, PDI, and *ζ*-potential, respectively. Then, a combination of surfactants (Kolliphor P188 and vitamin E-TPGS) at different concentrations was assessed. The resultant NPs showed an enhanced DLC (6.1 ± 1.1%) and EE (75.0 ± 13.0%) compared to the base formulation (Table 1), with other characteristics at optimal values (157.7 ± 7.7 nm, 0.16 ± 0.01, and −38.8 ± 2.1 mV) for size, PDI, and *ζ*-potential, respectively. When the mix of surfactants at the pH value of 5 was assessed altogether, both polymers (PLGA 50:50 and PLGA 75:25) showed improved DLC and EE (Table 1), moderate polydispersity, and an adequate size and surface charge. Similarly, an optimized formulation with PLGA 75:25 and the mix of surfactants (Kolliphor P188 and vitamin E-TPGS) at the pH value of 5 showed an increase in the DLC and EE values with regard to the base formulation, going from 6.0 to 6.7% of DLC and from 75.2 to 80.1% of EE, maintaining similar characteristics of size, distribution, and surface charge.

**FIGURE 2:**
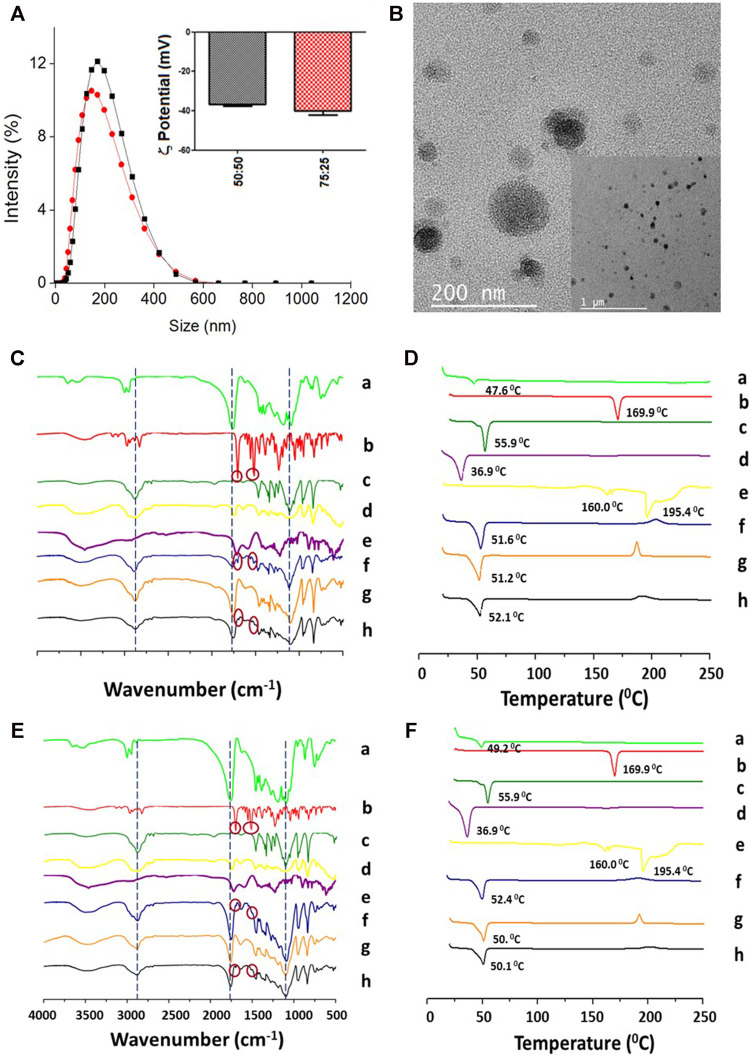
Physicochemical characterization of the PLGA-TPGS-pH5 (optimized formulation) NPs with encapsulated ITZ. **(A)** Hydrodynamic size and *ζ*-potential of PLGA 50:50 (black line and bars) and PLGA 75:25 (red line and bars). **(B)** Morphologic characterization of PLGA 50:50 NPs by TEM. **(C, D)** Characterization of PLGA 50:50 and **(E, F)** PLGA75:25 NPs, respectively, by FT-IR **(left)** and DSC **(right)**. PLGA (a), ITZ (b), Kolliphor P188 (c), vitamin E-TPGS (d), sodium citrate–cítric acid mixture (2:1) (e), physical mixture of 1:11:14.2:1.1 ITZ-to-PLGA-to–Kolliphor P188–to–vitamin E-TPGS weight ratio (f), and NPs from the mixture without (g) and with (h) encapsulated ITZ.

To investigate the interaction of ITZ with the components present in the NPs with optimized formulation, the chemical composition and physical changes of the materials were studied by FT-IR and DSC, respectively. Figures 2C,E show the FT-IR spectra of the individual precursors (a–e), the physical mixture (f), and NPs without (g) and with (h) encapsulated ITZ in both types of polymers. The physical mixture shows mostly the peaks at 1,767 cm^−1^ (C=O stretching) from the PLGA polymer, a little peak at 1,697 cm^−1^ (C=O stretching) from the amide group of ITZ and a small band of carbon–carbon double bonds at 1,511 cm^−1^, and at 2,844 cm^−1^ (C–H stretching) and 1,106 cm^−1^ (C–O stretching) from the stabilizer. The physical mixture pattern changed in the NPs with respect to empty NPs (e) and encapsulated ITZ (f), showing a peak at 1,760 cm^−1^ (C=O stretching) of higher intensity. The ITZ characteristic peaks at 1,697 cm^−1^(C=O stretching from the amide group), 1,520 cm^−1^ (C=N stretching), and 1,232 cm^−1^ (C–N stretching) could not be observed in NPs with encapsulated ITZ. Figures 2D,F show peaks of Tg and endothermal fusion peaks for PLGA polymers, ITZ, and Kolliphor P188 (a–c), as previously mentioned regarding the DSC analysis for the optimized formulation. Endothermal fusion peaks at 36.87°C for vitamin E-TPGS (d) and for the physical mixture of sodium citrate and citric acid (2:1) at 160 and 195.38°C (e) were observed. Besides, using the PLGA 75:25 polymer (Figure 2F), a unique phase transition was observed for the physical mixture (f), blank NPs (g), and encapsulated ITZ NPs (h) at 52.4, 50.0, and 50.1°C, respectively. A similar analysis is derived from the thermogram in Figure 2D for PLGA 50:50 optimized formulation (Yi et al., 2007).

### *In vitro* Release Kinetic Models

**FIGURE 3:**
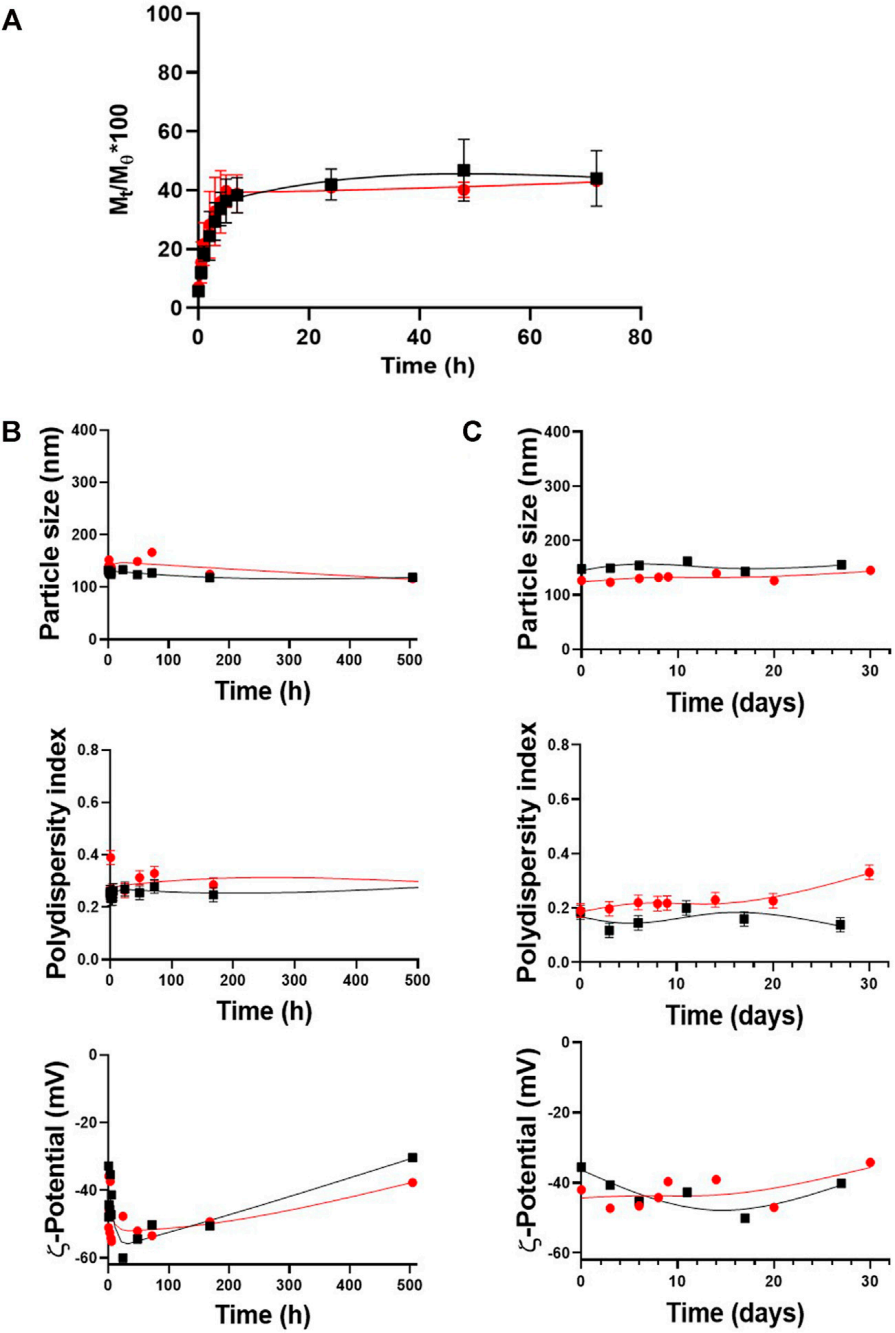
Release kinetics and stability of the optimized formulation upon time. **(A)** Release profiles for PLGA50:50-TPGS-pH5 and PLGA75:25-TPGS-pH5. Stability of PLGA50:50-TPGS-pH5 (black squares) and PLGA75:25-TPGS-pH5 (red circles) in terms of particle size, PDI, and *ζ*-potential **(B)** stored for one month at 4°C in an aqueous dispersion and **(C)** under the same kinetic release conditions (PBS with a pH value of 7.2, tween 1% v/v at 37°C). The error bars indicate the standard deviation of three successive measurements.

The ITZ release kinetics from the nanocarriers with the optimized formulation was evaluated in a release medium containing 1% v/v tween 80 to ensure infinite “sink” dilution conditions and emulate physiological conditions (PBS with a pH value of 7.2 at 37°C). The kinetics was determined by obtaining the fraction of ITZ released at a certain time (M_t_/M_θ_) for 72 h by HPLC. Figure 3A shows a kinetic profile of similar ITZ release, with a maximum ITZ release of 46 and 43% from the PLGA50:50-TPGS-pH5 and PLGA75:25-TPGS-pH5 nanocarriers, respectively, in agreement with reports in the literature (Ling et al., 2016; Varga et al., 2019). Data were adjusted using mathematical models searching for the best fitting by analyzing the correlation coefficient and other parameters related to the studied models (Supplementary Table S1) to inquire about the ITZ-release mechanism’s optimized formulation. It was found with regard to both types of particles that the release kinetics did not fit the zero-order, Higuchi, or Korsmeyer–Peppas models, as judged by their low correlation coefficients. The adjustment of the release profiles employing the Lindner and Lippold model to evaluate the “burst” effect presented relatively low values of correlation coefficients; therefore, the studied profiles did not fit this model either. However, when the systems were evaluated using the Ritger–Peppas and Peppas–Sahlin models, the correlation coefficients were closer to unity, showing a better fitting.

### Nanocarrier Stability

The stability of the PLGA-TPGS-pH5 optimized nanocarriers was evaluated in aqueous suspension at 4°C and under the release kinetics conditions, that is, PBS (pH 7.2), with 1% v/v tween at 37°C. Figure 3B shows that during the month of evaluation, aqueous dispersions of the formulations stored at 4°C presented high physical stability in terms of their particle size, with a variation of less than 5.5% of the relative standard deviation (RSD) (upper plot), moderate polydispersity (middle plot), and a medium-to-high negative potential (bottom plot). Similarly, Figure 3C shows the suspension under release kinetics conditions for both types of NPs. It demonstrated high colloidal stability during the 14 days of evaluation with particle size variations less than 10% of the RSD (upper plot), moderate polydispersity (middle plot), and medium-to-high negative potential (bottom plot). Due to PLGA75:25-TPGS-pH5 NPs presenting optimal characteristics (size, PDI, and *ζ*-potential) and higher DLC, EE, stability, and reproducibility, they continued to the *in vitro* studies.

### Antifungal Activity

**FIGURE 4:**
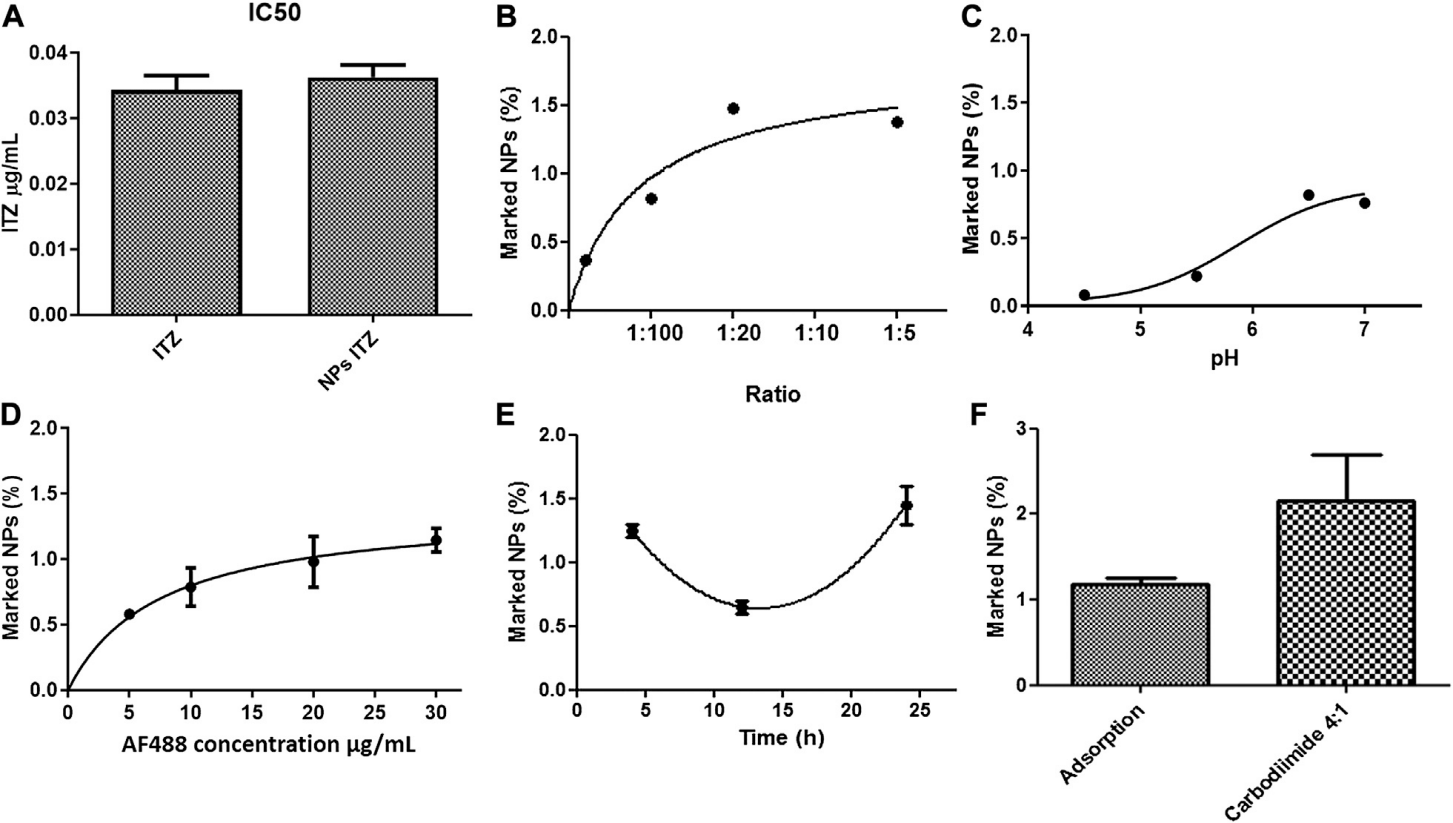
**(A)** IC50 of *Histoplasma capsulatum* treated with free itraconazole and itraconazole encapsulated into PLGA-TPGS-pH5 NPs. Effect of **(B)** pH, **(C)** antibody-to-NP ratio, **(D)** concentration of the secondary antibody, **(E)** incubation time in the adsorption of anti-F4/80 on NPs, and **(F)** evaluation of the functionalization method characterized by spectrophotometry (Ex: 490 nm–Em: 520 nm).

The minimum inhibitory concentration (MIC) of both free and encapsulated ITZ in the optimized nanocarriers was determined with a Colombian strain of *H. capsulatum* (CIB 1980). For this purpose, different concentrations ranging from 16 to 0.015 μg/ml of free and encapsulated ITZ in PLGA75:25 TPGS-pH5 NPs and empty NPs (as a control) were evaluated. The MIC was found to be 0.031 and 0.061 μg/ml for free and encapsulated ITZ using the macroscopic turbidity method (data not shown) and MTT assays (Supplementary Figure S3). The empty NPs at the high concentration (16 μg/ml) showed a slight inhibition of the fungal growth. The control with each component of NPs did not inhibit the fungal growth (data not shown). On the other hand, the mean inhibitory concentration (IC50) was estimated to be 0.031 μg/ml with both treatments (Figure 4A).

### Functionalization of Nanocarriers

The effect of pH, temperature, incubation time, and antibody-to-NP ratio of PLGA75:25 TPGS-pH5 NPs on the functionalization efficiency was evaluated by flow cytometry. It was observed that at pH 4.5 and 7.0, the NPs increased in size and PDI, suggesting agglomeration processes. At such extreme pH, peaks were generated in both negative and positive voltages, which could indicate the agglomeration and/or precipitation of non-absorbed and possibly denatured antibodies and/or the destabilization of NPs (data not shown). A significant decrease in *ζ*-potential was evident at 12 h, which could indicate a decrease in the stability of the NPs (data not shown). Overall, the optimal conditions for the adsorption method were 1:10 antibody-to-NP ratio (Figure 4B), a pH value of 6.5 (Figure 4C), and 24 h of incubation at 4°C (Figure 4F), increasing the size but maintaining a moderate polydispersity of 0.3 with a remained negative surface charge of −46 mV, indicating colloidal stability. A maximum extent of 1.48% of marked NPs was the highest value compared with samples functionalized at other conditions (Figure 4D). Furthermore, the optimal secondary antibody concentration was determined to be 20 μg/ml (Figure 4D).

For covalent coupling, available carboxyl groups were first semi-quantified through esterification with EDC/NHS, followed by FTIR analysis. Supplementary Figure S5A shows the FTIR spectrum of the esterified NPs (NP_PLGA_75: 25_Ester), unesterified NPs (NP_PLGA_75: 25), and EDC, NHS, and urea (which can be reaction residues) (Wang et al., 2011). The esterified NPs did not show the characteristic peaks of NHS, EDC, or urea, which indicates that the reaction was efficient and that the NPs’ washing process was adequate. Furthermore, esterified NPs showed a decrease in the area under the curve of the peak at *λ* = 3,492 cm^−1^, corresponding to the OH group, and an increase in the peak at *λ* = 1,758 cm^−1^, corresponding to the RC = O group. This fact is explained by the presence of the C= O attached to the cyclic chain. By measuring the areas under the curve of the esterified NPs (final areas) with respect to the control (initial areas) (Supplementary Figure S5C), the efficiency of the reaction could be estimated and related to the extent of activated carboxyl groups on the NP surface. About 40% of the carboxyl groups in NPs were available for activation and covalent antibody anchoring. The antibody’s coupling was assessed by the carbodiimide chemistry, obtaining 2.7% of functionalized NPs under the optimized conditions using the adsorption method based on protocols reported in the literature (Figure 4F).

### Formation of a Protein Corona

As an initial analysis of the protein corona formation at the outermost functionalized NP surface, depending on the functionalization method, they were incubated with 10–100% of FBS at 37°C for 1 h to simulate circulation conditions in the body. The nanoparticles modified using the adsorption method presented a larger size than NPs modified using the carbodiimide chemistry. With a concentration of 10% of FBS, the NPs of both methods tended to ±260 nm in size (Supplementary Figure S6A). The *ζ*-potential showed a significant decrease when modified using the adsorption method (only with 10% FBS), going from −58 to −37 mV, compared with that from covalent coupling that did not show a drastic change in the surface charge, going from −37.2 to −33.85 mV (Supplementary Figure S6B). Finally, when analyzing whether there was masking of the anti-F4/80 by the protein corona, there was no significant change in the anti-F4/80 functionalization efficiency under adsorption, varying from 0.92 to 0.98%. In contrast, the covalent coupling presented a significant decrease from 1.88 to 0.98% of anti-F4/80 at the NP surface (Supplementary Figure S6C).

### Specificity for Macrophages

**FIGURE 5:**
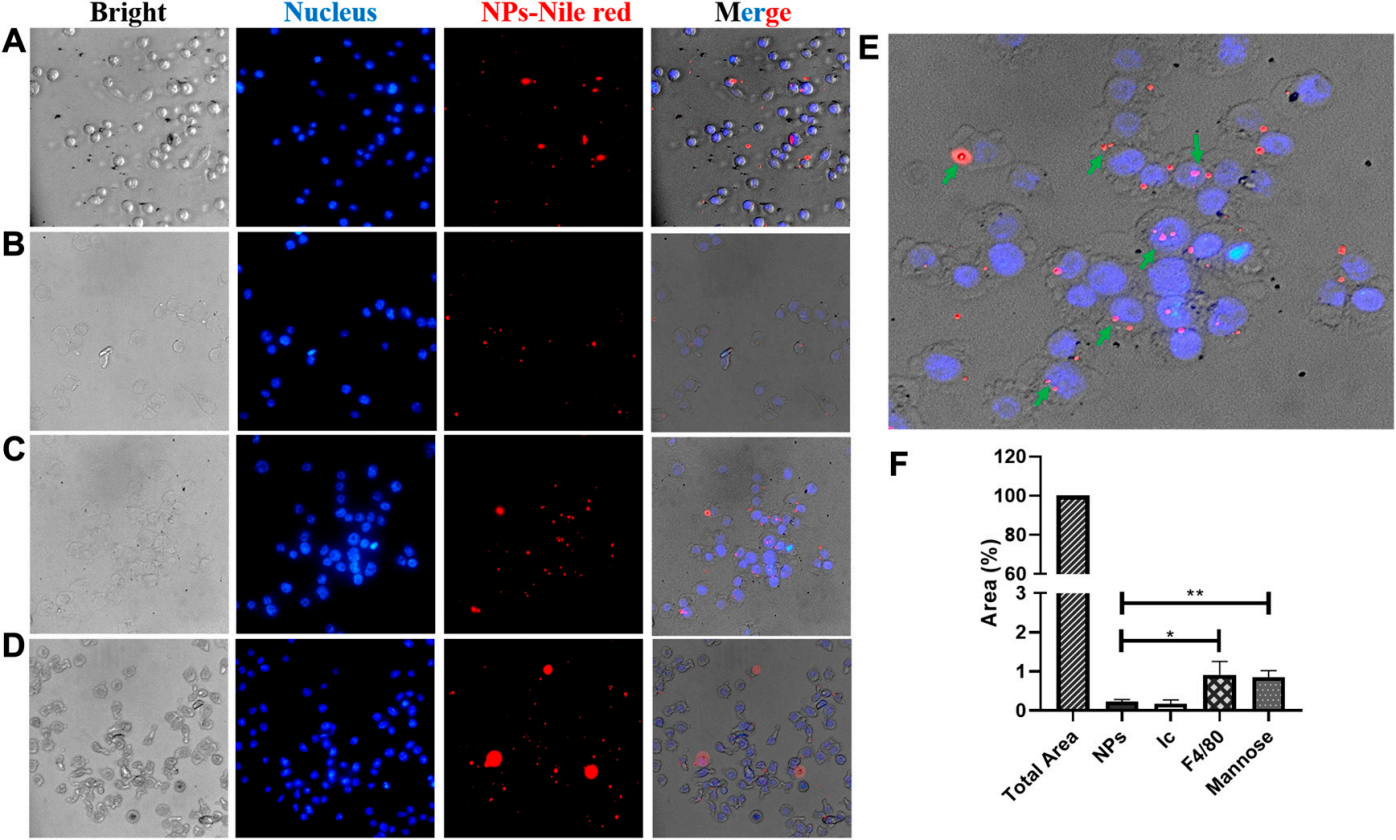
Fluorescence microscopy images. Endocytosis evaluation of functionalized NPs with Nile red encapsulated into the J774A.1 macrophage. NPs **(A)** without and **(B–D)** with different ligands, **(B)** control isotype, **(C)** F4/80 antibody, and **(D)** mannose, upon 3 h of incubation. Images are the bright field **(left)**, DAPI filter **(left-center)**, TRITC filter **(right-center)**, and merged **(right)**, respectively. The scale is 100 μm. **(E)** Enlarged image of the F4/80 merged image; green arrows indicate macrophages with endocytosed NPs. **(F)** Endocytosed NPs estimated by measuring Nile red by fluorescence intensity as described in the Materials and Methods section.

Nile red was used as a model of the hydrophobic compound to evaluate the functionalized NPs’ specificity due to its high hydrophobicity (solubility in water <1 μg/ml), relatively high molecular weight (318.37 g/mol), simple detection (UV–visible spectroscopy and fluorescence microscopy), and high photostability. Fluorescence microscopy was used to evaluate the specificity of functionalized NPs by J774A.1 macrophages. While NPs functionalized with the F4/80 antibody or D-mannose by covalent coupling were evaluated and compared, expected to present high specificity by macrophages, bare NPs and those functionalized with the IgG isotype were used as controls. The merged images show that F4/80 and D-mannose increased NP endocytosis (NPs—Nile red and blue cell nucleus) (Figures 5C,D), but bare NPs and NPs coated with the IgG isotype were less endocytosed by macrophages (NPs—Nile red and merged; Figure 5B). The uptake of NPs occurred in macrophages after 3 h of incubation, as shown in Figure 5. Nile red fluorescence was estimated as described in the Materials and Methods section to confirm the uptake differences among the differently functionalized NPs. In this fashion, the macrophage’s internalization extent was greater for those treated with F4/80 antibody–covered NPs (0.91%) than for those treated with D-mannose–functionalized NPs (0.84%), with bare (0.23%) and IgG isotype–coated (0.17%) NPs being used as controls, respectively (Figure 5F).

## Discussion

NPs for site-specific delivery of antifungal drugs to fight intracellular infections were developed to improve therapeutic efficacy. In this context, scientific literature shows that ITZ has been encapsulated into polymeric micelles by nanoemulsion but has relatively low DLC. Besides, most of them are not specific to macrophages, a principal target cell in infectious diseases and the main problem in developing antibiotic resistance. Functionalized NPs are a valuable option to regulate biodistribution through a target cell or tissue and can direct drugs across biological barriers (Scheme 1A) to a specific target through particle size modulation and surface modification with ligands to increase cell penetration and achieve specificity of action. By using this strategy, NPs can be directed to the infection foci, where the main burden of pathogens is found to release its cargo. In addition, higher drug doses at the infected site can be administered (Scheme 1A), diminishing existing microorganism resistance or eliminating microorganisms, thus causing fewer adverse effects on patients.

In this study, NPs’ properties were tuned to encapsulate an optimal ITZ concentration in a highly biocompatible system. With the base formulation, when studying the influence of the PLGA-to-ITZ ratio on the physicochemical properties of NPs prepared by nanoemulsion, the system containing 7.5 mg of ITZ produced a larger particle size, which increased as relative viscosity increased, in agreement with reports in the literature (Wooster et al., 2008; Gupta et al., 2016). Furthermore, a 1:5 ITZ-to-PLGA ratio produced NPs with a higher polydispersity (PDI = 0.41) than a 1:10 ratio (PDI = 0.19), as reported elsewhere (Bian et al., 2013). This effect may be related to the inadequate adsorption of surfactant at a lower ITZ-to-PLGA ratio and a higher amount of ITZ at the surface level that did not effectively coat the emulsified droplets, causing their destabilization (Bian et al., 2013; Lakkireddy and Bazile, 2016; Wilkosz et al., 2018) and further agglomeration. Furthermore, a 1:11 ITZ-to-PLGA ratio produced NPs with higher DLC values (4.0 ± 0.3%) but lower EE values (47.6 ± 3.2%). The higher molecular weight of PLGA 50:50 possibly increased its hydrophilic character, avoiding an adequate partitioning of ITZ in the NPs by hydrophobic and electrostatic PLGA–ITZ interactions so that ITZ might be migrating toward the continuous phase during the nanoemulsion process, producing lower EE. However, such PLGA 50:50 NPs’ base formulation presented optimal size (188.5 ± 3.0 nm), PDI (0.23 ± 0.04), and *ζ*-potential (−39.9 ± 2 mV), compared to other ratios evaluated (1:10 and 1:25) (Figure 1; Table 1). The smaller average particle size of PLGA 75:25 NPs compared to that of PLGA 50:50 may be related to the lower molecular weight of PLGA 75:25, which decreases the system’s viscosity. In contrast, the increased DLC may be explained by the higher hydrophobic interaction between the aliphatic carbons of the ITZ and the methyl groups of PLGA 75:25, which resulted in higher stabilization of the active principle within NPs. In other words, higher PLGA 75: 25–ITZ hydrophobic interactions produced higher packaging and, therefore, smaller nanostructures.

The next set of experiments introduced some base formulation modifications to improve its physicochemical and structural properties, evaluated by the DLC, EE, and drug-release profile. Modifications consisted of lowering the pH of the aqueous solution to five and including a multipurpose amphiphilic excipient such as vitamin E-TPGS in a 1:10 ratio by weight with respect to PLGA, one at a time. Such a slightly acidic medium increased ITZ ionization as its amide carbonyl group (R–N–C=O– Hδ^+^) was protonated, thereby increasing its solubility and dispersibility (Prentice and Glasmacher, 2005; Kapoor et al., 2015; Alhowyan et al., 2019). Therefore, the higher amount of stabilizing electrostatic interactions among the formulation components decreased ITZ diffusion toward the continuous phase during the nanoemulsion process to avoid considerably affecting PLGA composition but impacting the enhanced DLC and EE. Then, a combination of surfactants (Kolliphor P188 and vitamin E-TPGS) at different concentrations was assessed. Unlike the base formulation, Kolliphor P188 was added in a lower concentration (9.37 mg/ml) and dissolved into the aqueous phase, while vitamin E-TPGS was used in a 1:10 ratio of PLGA 75:25 to vitamin E-TPGS and dissolved into the organic phase. This formulation increased DLC and EE, and such enhancement may be explained by increased hydrophobic interactions of the aliphatic tail (18 carbons) from vitamin E-TPGS with the aliphatic carbons of ITZ in addition to electrostatic interactions of the carbonyl ester group from vitamin E-TPGS with the triazole groups of ITZ. PLGA 75:25 NPs’ DLC (6.7 ± 1.3%) was slightly higher than PLGA 50:50 NPs’ DLC (6.4 ± 0.7%), and this PLGA 75:25–based formulation went to the next set of experiments. On the other hand, it is known that high Kolliphor P188 concentrations might be involved in the production of free radicals and reactive oxygen species during the sonication process when NPs are assembling, thus generating biological responses such as affectation of mitochondrial respiration and ATP synthesis, among other adverse effects, when tested *in vivo* (Batrakova and Kabanov, 2008; Wang et al., 2012). Therefore, it is important to highlight that through this improved formulation, the content of Kolliphor P188 was reduced more than 2.5-fold with respect to the base formulation, thereby expecting higher biocompatibility.

FT-IR and DSC results for both formulations (base and optimized) showed the polymer–drug interactions in the ITZ-encapsulated NPs. In this context, these interactions occur possibly due to a complex formed between the triazole (or amine) group of ITZ and the carboxylic group from the PLGA hydrophobic tail, as pointed out by Yi and collaborators (Yi et al., 2007). Additionally, the polymer’s characteristic band at 1,745 cm^−1^ (C=O stretching) in the NPs with ITZ was highly intense. This band is indicative of carboxylic end groups available for further functionalization with ligands. Remarkably, the melting peak from ITZ completely disappeared in the NP-encapsulated ITZ thermogram, indicating the absence of the crystalline state of the encapsulated drug. The interaction among the formulation precursors allows a certain freedom of molecular movement to organize themselves in a crystalline way, reflected by small exothermic peaks of low intensity between 150 and 210°C for the physical mixture and the empty and loaded NPs in the two types of nanocarriers.

TEM images determined structural and morphological characteristics of the NPs from the two PLGA polymers with optimized formulation (Figures 2B; Supplementary Figure S2), showing spherical core–shell–like structures. NPs consist of the PLGA hydrophobic core and the hydrophilic shell from the hydrophilic tails of the Kolliphor P188 and vitamin E-TPGS surfactant mixture. Schemes 1A,B illustrate a diagram representing the composition of core–shell–like NPs and the corresponding receptor–ligand interactions in the cellular surface to accomplish internalization processes (Lin et al., 2010). Steric repulsion made by hydrophilic polymer chains can improve nanocarriers’ circulation time in the blood by overpassing the phagocytic mononuclear system (Mu and Feng, 2003; Mu and Feng, 2003). The particle size of PLGA 75:25 NPs, estimated by TEM, was smaller than that of PLGA 50:50 NPs for the optimized formulations and comparable with those obtained from DLS (Table 2). Moreover, the particle size by DLS was overestimated, as expected.

**TABLE 2:** Comparison of average particle size by DLS and TEM.

Polymer (PLGA)	Size (nm), DLS	Size (nm), TEM
50:50	188.5 ± 3.0	145.0 ± 28.2
75:25	150.4 ± 6.4	132.0 ± 36.6
50:50-TPGS-pH5	165.0 ± 9.7	140.3 ± 26.7
75:25-TPGS-pH5	147.3 ± 7.7	131.0 ± 30.8

ITZ release kinetics from the nanocarriers with the optimized formulation was studied. Although it was expected that the more hydrophilic PLGA50:50-TPGS-pH5 NP system would degrade faster than the PLGA75:25-TPGS-pH5 ones, the fact of similar behaviors being present in both types of nanocarriers may be explained by the higher molecular weight of the PLGA 50:50 polymer, which has a higher amount of hydrophobic interactions with ITZ, comparable to the hydrophobic interactions of PLA in the PLGA 75:25 of lower molecular weight. In other words, the higher molecular weight of PLGA 50:50 impacts the release kinetics more than the hydrophilic character of this polymer as compared to PLGA 75:25 of lower molecular weight (Fredenberg et al., 2011). Furthermore, the multipurpose behavior of vitamin E-TPGS as a matrix component allows the establishment of electrostatic and hydrophobic interactions within the nanoparticle that equates the two types of systems with different chemical natures. In both types of particles, it was found that the release kinetics does not fit the zero-order, Higuchi, or Korsmeyer–Peppas models if it is judged by the low correlation coefficients obtained. The fact that it does not follow a zero-order model allows us to ensure that the ITZ release rate is not constant over time and that the polymer chains’ relaxation does not control the release process. As it does not follow a kinetic model like Higuchi or Korsmeyer–Peppas, it implies that the swelling/contraction phenomena must be taken into account. Release kinetics fit better with the Ritger–Peppas and Peppas–Sahlin models. It is speculated that there is a coupled mechanism or a superposition of apparently independent mechanisms such as Fickian diffusion and the polymeric matrix’s swelling/relaxation. It is important to highlight that the Fickian mechanism’s contribution (K1) is greater than the contribution of the polymer chains’ relaxation mechanism (K2), as indicated by the higher value of K1 compared to K2. Furthermore, in both types of NPs, the *n* value was very close but higher than 0.5, indicating a quasinormal Fickian diffusion (Bruschi, 2015).

The PLGA-TPGS-pH5 formulations stored at 4°C presented high physical stability. By decreasing the temperature, the kinetic and diffusive energy and the collision frequency among the NPs decreased accordingly, and therefore, particle aggregation was less. Similarly, Figure 4C shows the suspensions under release kinetics conditions for both types of NPs. It demonstrated high colloidal stability during the 14 days of evaluation with particle size variations less than 10% of the RSD (upper plot), moderate polydispersity (middle plot), and medium-to-high negative potential (bottom plot). These results support the hypothesis that the ITZ-release kinetic profile has a low contribution caused by hydrolytic erosion/degradation of the PLGA matrix.

By evaluating MICs with the different free- and encapsulated-ITZ treatments in PLGA-TPGS-pH5 NPs, it was possible to show that encapsulated ITZ preserved its antifungal activity against the fungus requiring a higher concentration of 0.061 μg/ml with respect to 0.031 μg/ml of free ITZ. However, when the IC50 of both treatments was calculated, it was evident that the same amount of free or encapsulated ITZ was needed to obtain 50% control of the fungus (Figure 4A). These results are related to the type of kinetic release that the NPs presented (Figure 3A), where after 72 h, only 43% of the encapsulated ITZ was released. Therefore, to inhibit 100% of the fungal growth, a higher concentration of encapsulated ITZ would be necessary than the free one, but the amount of the ITZ released from the NPs was high enough to inhibit 50% of the fungal growth. The concentration of ITZ in the assay with NPs may be achieving the maximum saturation point, hindering the diffusion of more ITZ from the hydrophobic NPs’ core to the hydrophilic phase. Furthermore, the empty NPs at the higher concentration (16 μg/ml) presented an inhibitory action against the fungal growth, which was not related to the NP components considering the controls’ results, where they did not show any inhibition. Therefore, the inhibition might be related to the fact that NPs at high concentrations can bind easier to proteins or other fungus molecules, affecting their growth.

Methods for functionalizing NPs have a strong influence on the NPs’ performance with regard to their specificity for target cells. They can influence the amount and orientation of ligands coupled onto the NPs, improving the uptake of NPs for the macrophages. When comparing the methods, we observed that the chemical coupling presented a higher degree of functionalization than the physical adsorption one, being more reproducible and maintaining the stability of the NPs. Furthermore, results from Supplementary Figure S6 show that the NPs functionalized by covalent and adsorption coupling in the presence of 10% FBS had an insignificant decrease in the antibody’s coating extent, indicating low masking of the ligands for both methodologies. On the contrary, the presence of 100% FBS, functionalized by covalent coupling, caused a significant decrease in the antibody’s coating extent, indicating more effective masking with regard to those modified by adsorption. However, this effect may be related to the higher amount of the ligand in the NPs with covalent coupling than in the NPs coated by adsorption, suggesting the formation of the more extensive protein corona on the NPs as reported in the literature (Tonigold et al., 2018).

Regarding evaluating the specificity of functionalized NPs, results showed that NPs functionalized with the F4/80 antibody and mannose using the covalent method increased their endocytosis into macrophages significantly. Therefore, we demonstrated that NP functionalization might increase the number of endocytosed NPs, depending on the type of (bio) molecule coating. In this sense, it is well known that different macrophage populations exist, characterized by their heterogeneity, plasticity, and expression of diverse receptors. For example, lung macrophages in mice (alveolar macrophages) have high expression of mannose and the siglec-F receptor and low expression of the F4/80 receptor as compared to peritoneal macrophages that present an intermediate expression of F4/80, low expression of mannose, and no expression of the siglec-F receptor (Gordon and Plüddemann, 2017). Additionally, some reports have demonstrated that mannose-functionalized NPs controlled leishmaniasis infection by increasing the distribution of the functional NPs in the selected organs such as the liver and spleen and decreasing the amount in the peripheral blood as compared to NPs without mannose (Asthana et al., 2015; Barros et al., 2015). This is the first report showing that functionalization of NPs with F4/80 antibodies can help to improve macrophage-targeted therapy and with similar efficiency to that of mannose-coupled NPs. Therefore, F4/80-functionalized NPs open up the possibility of use in therapies directed toward other subpopulations of macrophages that do not present a high expression of mannose, that is, peritoneal macrophages (Hussell and Bell, 2014). Overall, functionalized NPs are a versatile technological platform that might be extended to a broad spectrum of applications for the treatment of intracellular infectious diseases.

## Conclusion

We successfully encapsulated ITZ into core–shell–like NPs based on two types of PLGA, obtaining stable and moderately polydisperse nanocarriers with adequate size and optimal DLC (6.6%) and EE (80%) by lowering the pH and by modulating the type and concentration of a mixture of surfactants. Whereas FT-IR and DSC analysis demonstrated the ITZ–PLGA interactions, FT-IR showed the presence of carboxylic end groups available to react with ligands. The release profile of PLGA 75:25 and PLGA 50:50 NPs fitted well with the Fickian diffusion model. The NPs showed stability in water at 4°C and under release kinetics conditions. Encapsulated ITZ efficiently eliminated *H. capsulatum*, with a similar IC50 to that of free ITZ. The covalent coupling to functionalize the NPs was more efficient than the adsorption method, but the protein corona masking was similar in both methods. *In vitro* assays showed that the NPs functionalized with F4/80 and mannose increased the uptake of NPs by J774 macrophages. Therefore, the F4/80-coupled NPs can be an alternative for tagging other subpopulations of macrophages*.* Due to the multiple mechanisms presented by pathogens that cause intracellular infections, the use of functionalized NPs would allow a much more specific treatment of these infections, reducing undesired effects in patients. In this context, current research on macrophage target therapies is directed toward finding different types of ligands for targeted drug release into specific macrophage subpopulations.

## Data Availability

The original contributions presented in the study are included in the article/Supplementary Material; further inquiries can be directed to the corresponding author.
