# Supplementary material for: Functional Nanocarriers for Delivering Itraconazole Against Fungal Intracellular Infections
Source: Front Pharmacol. 2021 Jun 28;12:685391. doi: 10.3389/fphar.2021.685391 (PMC8274696; doi:10.3389/fphar.2021.685391)
Supplement: Supplementary file 1 [file DataSheet1.docx]

Functional nanocarriers for delivering itraconazole against fungal intracellular infections.

Susana P. Mejía^1,2^, Arturo Sánchez^1^, Viviana Vásquez^1^, Jahir Orozco^1*^

^1^Max Planck Tandem Group in Nanobioengineering, University of Antioquia, Complejo Ruta N, Calle 67 Nº 52-20, Medellín 050010, Colombia

^2^ Experimental and Medical Micology Group, Corporación para Investigaciones Biológicas (CIB), Medellín, Colombia

Supplementary Material

**Table S1.** Kinetic models of ITZ-release profiles from PLGA50:50-TPGS-pH5 and PLGA75:25-TPGS-pH5 nanocarriers.

| **Model** | **Parameters**  **PLGA 50:50-TPGS-pH5** | **Parameters**  **PLGA 75:25-TPGS-pH5** |
| --- | --- | --- |
| **Zero Order**  M_t_/M_θ_= Kt | K= 0.9554 ± 0.4085  R^2^ = 0.897 | K = 0.7891 ± 0.4082  R^2^ = 0.2811 |
| **Higuchi**  M_t_/M_θ_= Kt^1/2^ | K= 6.8146 ± 1.7995  R^2^ = 0.6559 | K = 5.8653 ± 1.8903  R^2^ = 0.5521 |
| **Korsmeyer-Peppas**  M_t_/M_θ_= K_1_t^n^ | K_1_ = 21.4528 ± 2.2070  n = 0.291 ± 0.0493  R^2^ = 0.8001 | K_1_ = 24.8832 ± 2.3713  n = 0.2051 ± 0.0476  R^2^ = 0.7410 |
| **Ritger-Peppas**  M_t_/M_θ_= Kt^1/2^ +K_2_t | K_1_ = 21.8604 ± 0.4777  K_2_ = -2.6961 ± 0.1249  **R^2^ = 0.9865** | K_1_ = 24.8175 ± 0.1649  K_2_ = -3.3283 ± 0.0431  **R^2^ = 0.9981** |
| **Peppas-Sahlin**  M_t_/M_θ_= K_1_t^n^ +K_2_t^2n^ | K_1_ = 19.9449 ±0.3560  K_2_ = -2.1592 ± 0.0914  n = 0.5638 ± 0.0105  **R^2^ = 0.9981** | K_1 =_ 24.6048 ± 0.3691  K_2_ = -3.2585 ± 0.1160  n = 0.5057 ± 0.0087  **R^2^ = 0.9979** |


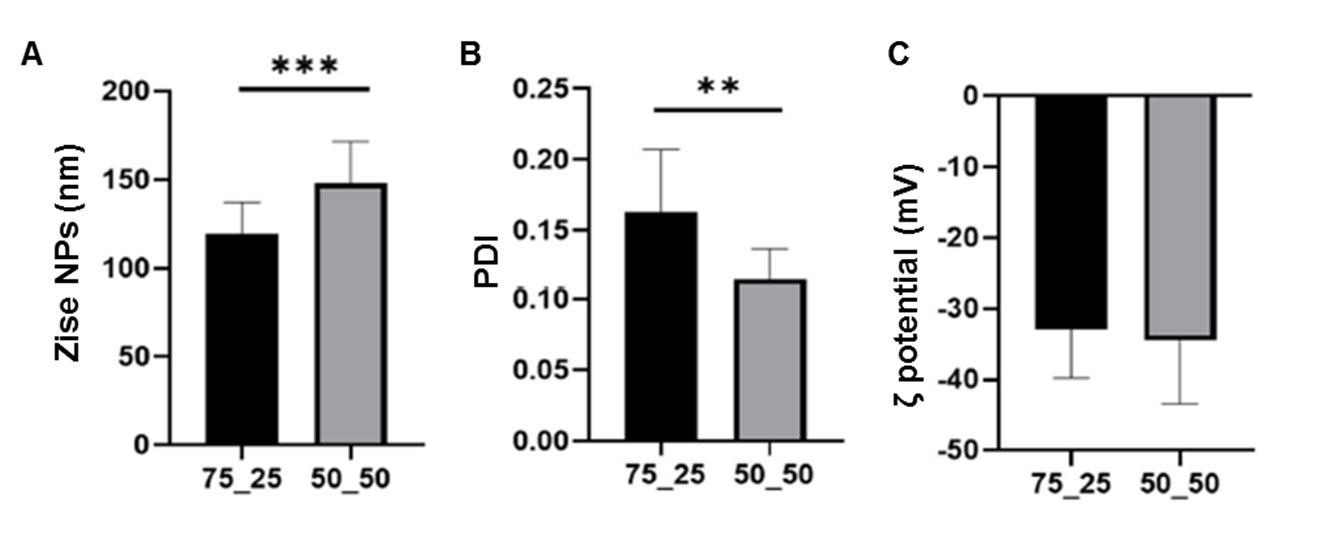


Figure S1. Comparison of size (A), size distribution (B) and surface charge (C) of nanoparticles from PLGA50: 50-NPs and PLGA75: 25-NPs with encapsulated Nile red. ** and *** indicate statistically significant differences with p between 0.01 and 0.001 and p < 0.001, respectively.


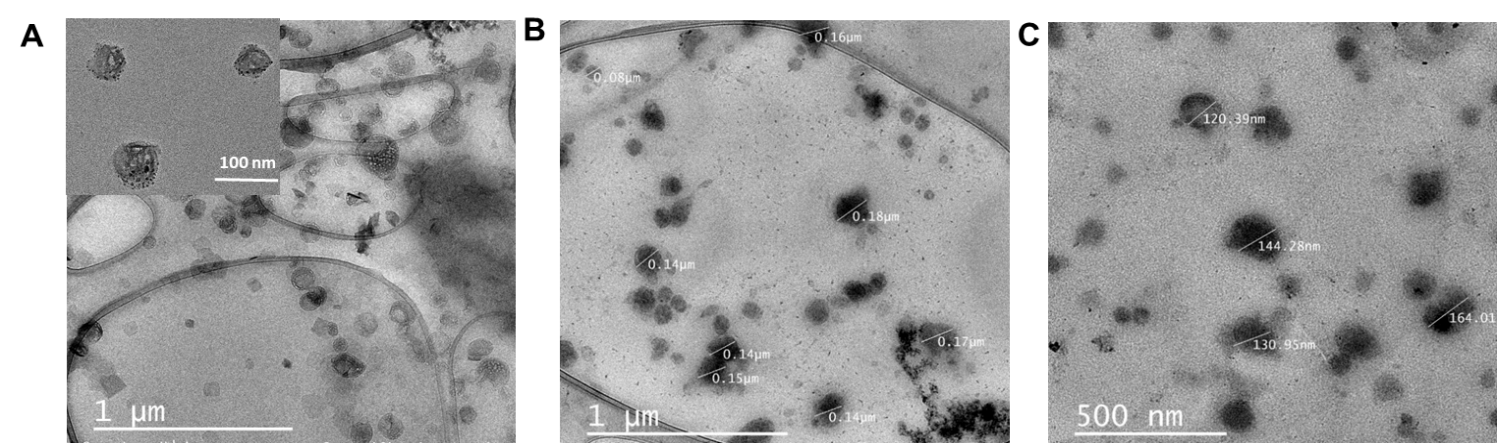


**Figure S2.** Morphologic characterization by TEM of PLGA NPs base formulation with the polymer PLGA 75:25 (A), and optimized formulation (PLGA-TPGS-pH5) in two scales B) 1µm and C) 500 nm. (Reviewer # 1, Section Q3- question No.2 and 3).

**A**

**B**

**
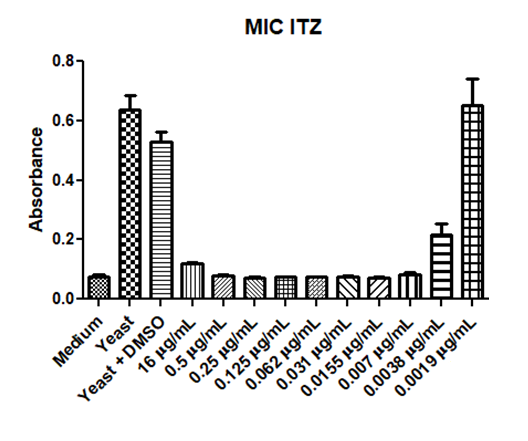

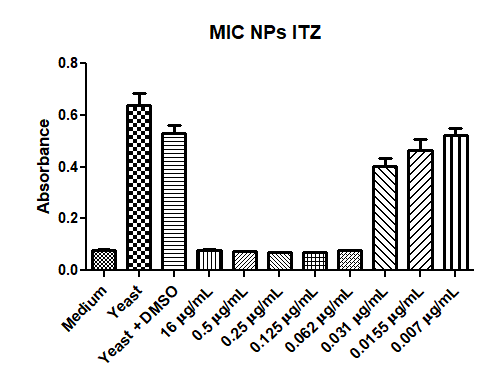
**

**
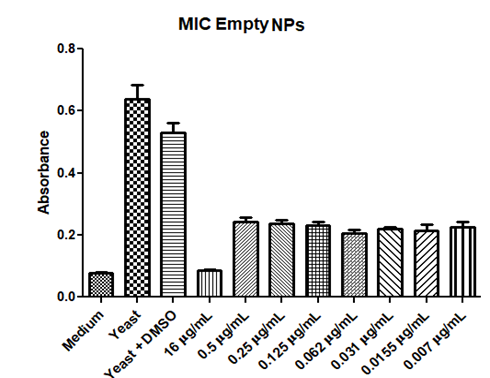
**

**C**

**Figure S3. A)** MIC of free Itraconazole (ITZ) B) Itraconazole encapsulated into PLGA75:25 TPGS-pH5 NPs C) and empty PLGA75:25 TPGS-pH5 NPs on *H. capsulatum* CIB1980 strain.


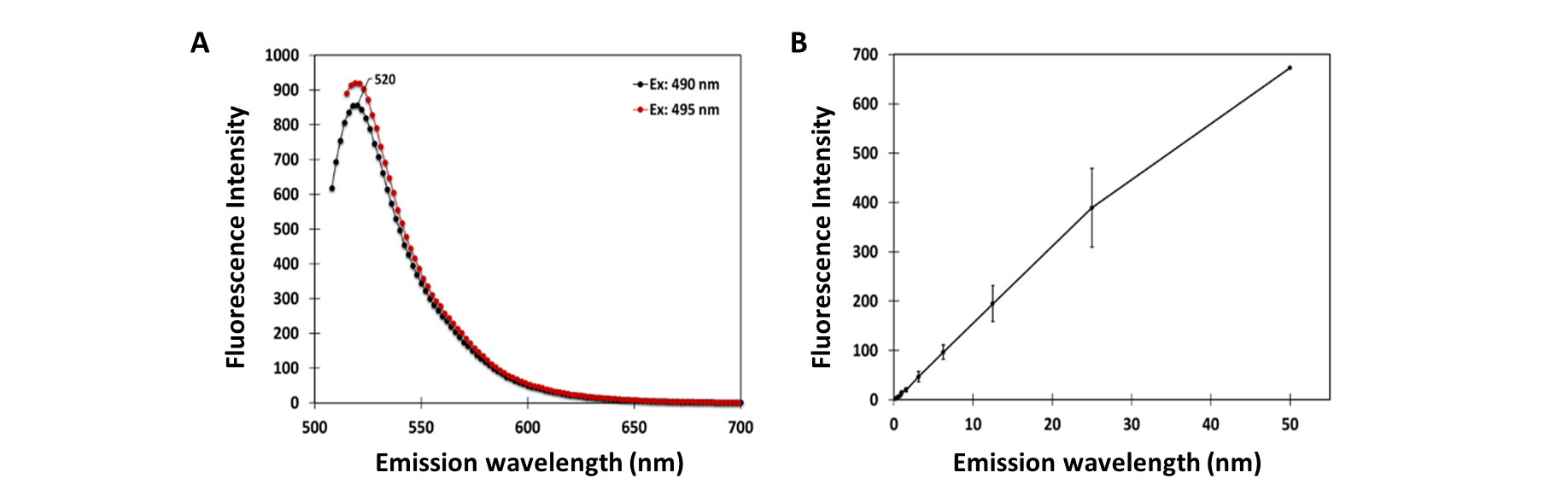


**Figure S4.** A) Fluorescence spectra of AF488 with excitations at 490 nm and 495 nm B) Calibration curve of AF488 (Ex: 490 nm, Em: 520 nm).

**
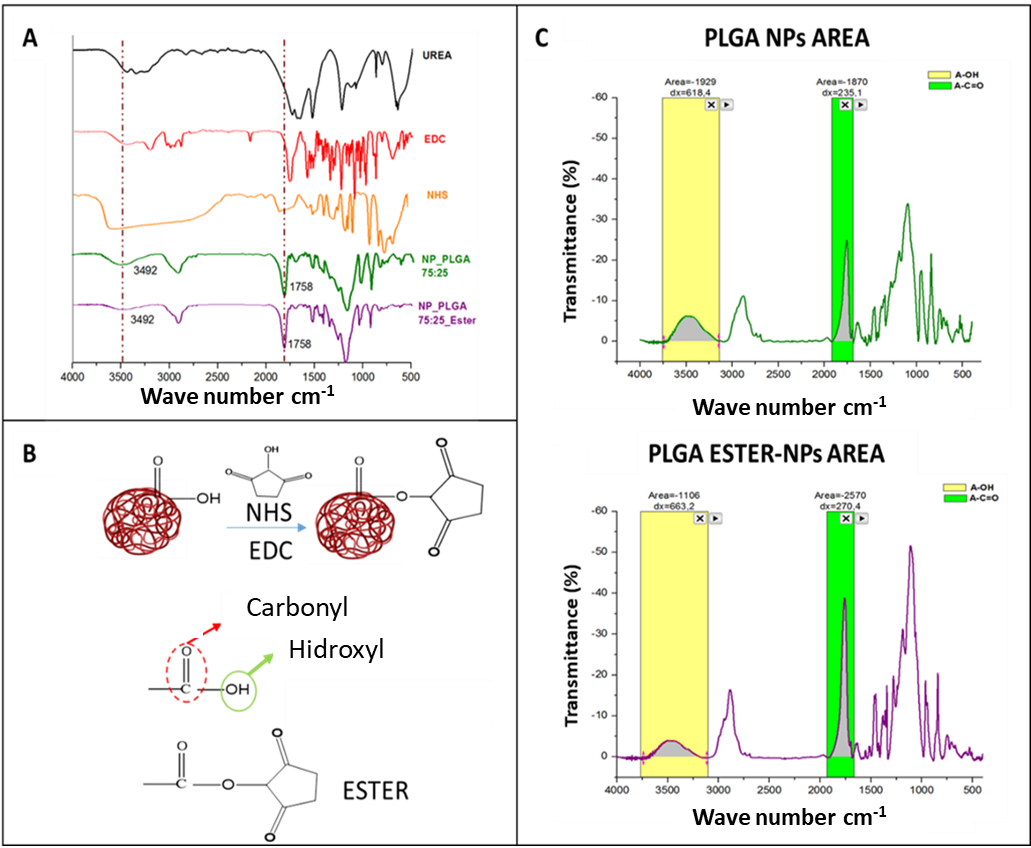
**

**Figure S5** Reviewer # 1, Section Q3- question No.4)**.** A) FTIR spectrum of all components of the reaction and by-products. B) The area under the curve of the esterified and control NPs spectra and C) Esterification reaction on NPs.

**
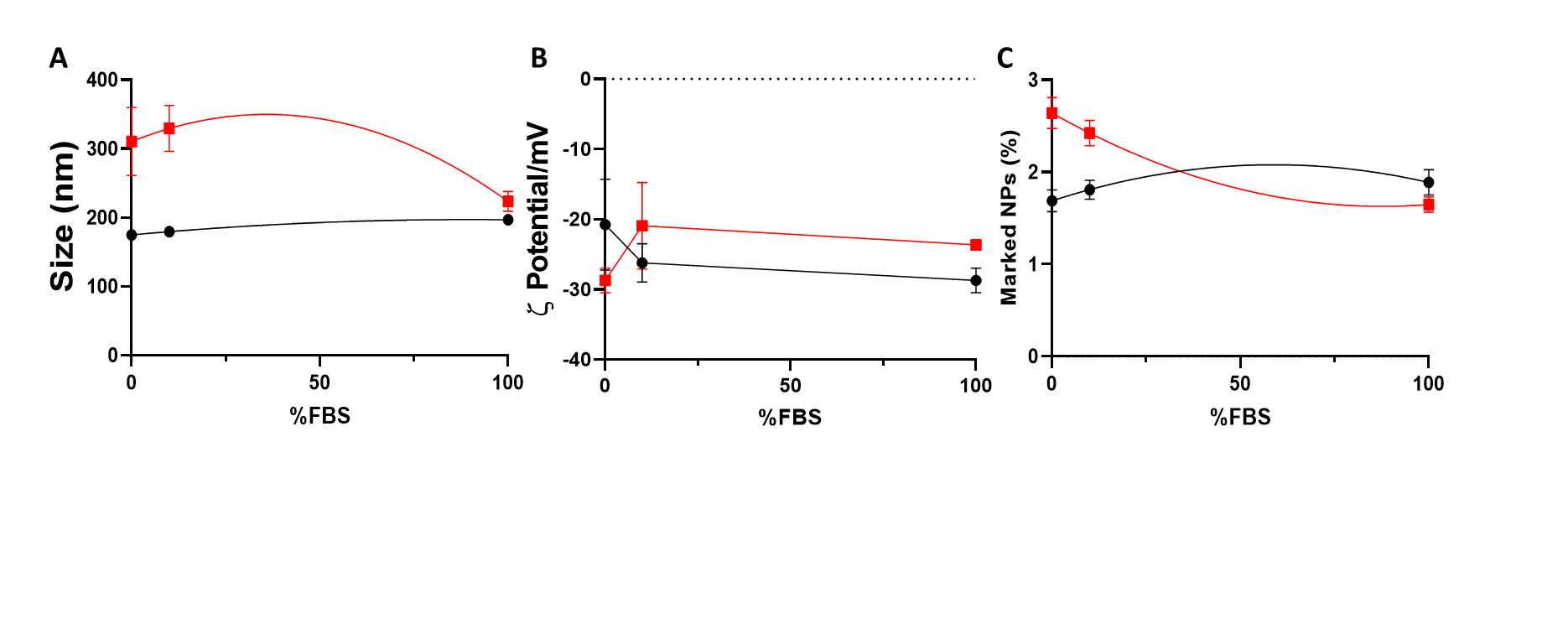
**

**Figure S6.** Evaluation of the effect of the protein corona over A) size B) ζ-potential and C) the accessibility of immobilized anti F4/80 on NPs by adsorption (black line) and covalent coupling (red line) methods, at concentrations of 0, 10 and 100 % fetal bovine serum (FBS).
